# Evaluation of interleukin-32 and cyclooxygenase-2 expression in HAM/TSP patients and HTLV-1 asymptomatic carriers

**DOI:** 10.22038/ijbms.2021.50821.11569

**Published:** 2021-07

**Authors:** Niayesh Hatatian, Reza Bosstani, Asadollah Mohammadi, Saeedeh Mehraban, Maryam Mahdifar, Fariba Zemorshidi, Sayed-Hamidreza Mozhgani, Ali Haji Ghadimi, Mohsen Foroughipour, Houshang Rafatpanah

**Affiliations:** 1 Immunology Research Center, Inflammation and Inflammatory Diseases Division, Faculty of Medicine, Mashhad University of Medical Sciences, Mashhad, Iran; 2 Department of Neurology, Faculty of Medicine, Mashhad University of Medical Sciences, Mashhad, Iran; 3 Cellular and Molecular Research Center, Research Institute for Health Development, Kurdistan University of Medical Sciences, Sanandaj, Iran; 4 Department of Microbiology, School of Medicine, Alborz University of Medical Sciences, Karaj, Iran; 5 Shahid Beheshti University of Medical Sciences, Tehran, Iran

**Keywords:** Cyclooxygenase-2, HAM/TSP, HTLV-1, Interleukin-32, Inflammation

## Abstract

**Objective(s):**

HTLV-1 associated myelopathy/tropical spastic paraparesis (HAM/TSP) is a neuroinflammatory disorder associated with HTLV-1. Cytokines and inflammatory mediators have a major role in forming inflammation in HAM/TSP patients. This study aimed to measure the levels of IL-32, a proinflammatory cytokine associated with autoinflammatory disorders, and also cyclooxygenase -2 (COX-2) as a key mediator of inflammatory pathways in HAM/TSP patients and HTLV-1 asymptomatic carriers (ACs).

**Materials and Methods:**

Peripheral blood monocyte cells (PBMCs) were isolated from HAM/TSP patients, ACs, and healthy controls (HCs), and DNA and RNA were extracted to evaluate HTLV-1 proviral load (PVL) and expression of IL-32 and COX-2, using real-time PCR. Serum levels of IL-32 were determined by using an ELISA assay.

**Results:**

The expression level of IL-32 was significantly higher in ACs compared with HAM/TSP patients and HCs (*P*<0.0001 and *P*>0.05, respectively). There were no statistically significant differences in the expression levels of Cox-2 and protein levels of IL-32 between the study groups. HTLV-1 PVL was higher in HAM/TSP patients compared with ACs.

**Conclusion:**

Results showed increased mRNA levels of IL-32 in ACs. Since HTLV-1 PVL in ACs is lower than in HAM/TSP patients, it could be concluded that IL-32 might be an HTLV-1 inhibitor that seems to control virus replication. Despite the difference in IL-32 mRNA levels between study groups, no statistically significant differences were observed in IL-32 serum levels. Also, there were no significant differences in COX-2 expression.

## Introduction

Human T cell leukemia virus type 1 (HTLV-1), the first human oncoretrovirus to be discovered, is endemic in different geographical regions such as Southwestern Japan, Western Africa, Central and South America, the Caribbean islands, the Melanesian Islands, and the Middle East and infects about 10–20 million people worldwide (1, 2). Mashhad, the capital of Razavi Khorasan province in the northeast of Iran was reported as an endemic region for HTLV-1 infection in 2012. The prevalence of HTLV-1 infection in Mashhad was estimated to be 2.1% among the general population (3).

HTLV-1 is the etiological agent of adult T cell leukemia (ATL), a malignancy of mature T cells, and HTLV-1 associated myelopathy/tropical spastic paraparesis (HAM/TSP), a disabling neuroinflammatory disease that impacts patient’s quality of life (4, 5).

HAM/TSP results in demyelination of the spinal cord and the patients suffer from muscle weakness and hyperreflexia of the lower limbs, sensory disorders, urinary incontinence, impotence, and constipation (6).

In HAM/TSP, increased production of inflammatory cytokines such as interferon gammas (IFN-γ) and tumor necrosis factor-alpha (TNF-α) along with a reduction in Th2 cytokines including interleukin (IL)-4 and IL-10 can lead to development of neuroinflammation (7). Proinflammatory cytokines, which play a major role in the pathogenesis of HTLV-1 associated diseases, have been identified in HTLV-1 infected cells (8).

Interleukin-32 is a proinflammatory cytokine expressed by natural killer cells (NKCs), T lymphocytes, blood monocytes, and epithelial cells and can induce several proinflammatory cytokines such as IL-1β, IL-6, IL-8, and IFN-γ (9). Furthermore, elevated IL-32 levels play important roles in viral infections such as human papillomavirus (HPV), hepatitis C virus (HCV), hepatitis B virus (HBV), Epstein bar virus (EBV), and human immunodeficiency virus (HIV) infections (10-14). Cyclooxygenase 2 (COX-2) is one of the main factors that regulate IL-32 production in response to viral infection and this inflammatory mediator is stimulated by cytokines such as IL-17 (10). COX-2 is a membrane-bound enzyme that plays a critical role in the biosynthesis of prostaglandins which regulate the inflammatory response (15). COX-2 is an inducible form of cyclooxygenase that is expressed in specific cells including activated macrophages, monocytes, and lymphocytes, in response to inflammatory stimuli such as proinflammatory cytokines, resulting in up-regulation of gene expression and induced local inflammation (16). In several chronic and autoimmune diseases, COX-2 is overexpressed and plays an important role in disease pathogenesis (16, 17). 

Since proinflammatory cytokines play an important role in neuroinflammation, the present study was conducted to evaluate the levels of IL-32 and COX-2 and their correlation with HTLV-1 proviral load (PVL) in HAM/TSP patients and HTLV-1 ACs to see whether these two factors are associated with disease progression in HAM/TSP. The association of cytokines with HAM/TSP pathogenesis could be used to develop therapeutic approaches to control disease progression

## Materials and Methods

***Study population***

The present study included three groups of individuals: HAM/TSP patients who were serologically positive for HTLV-1 (ELISA with confirmation by PCR) with clinical and neurological symptoms that were diagnosed by a neurologist, ACs who were HTLV-1 seropositive with no clinical symptoms, and the seronegative healthy control (HC) group. Study groups were individually matched on age and sex. All participants were checked for other viral infections such as human immunodeﬁciency virus (HIV), hepatitis B (HBV), and hepatitis C (HCV), and positive cases were excluded from the study. Participants with a history of hypertension, diabetes, coronary heart disease, atherosclerosis, kidney disease, autoimmune diseases, a history of treatment with corticosteroids or other immunosuppressive drugs over the past year were also excluded (18). Each study group contained 21 individuals. The HAM/TSP patients and ACs were enrolled in the present study from cases referred to the HTLV-1 clinic, Qaem hospital, Mashhad University of Medical Sciences (MUMS), Mashhad, Iran, and written informed consent was obtained from all participants. The ethics committee of Mashhad University of Medical Sciences, Mashhad, Iran, approved the study.

***PBMC isolation and nucleic acid extraction***

Six milliliters of blood were collected from each participant in sterile vacutainer EDTA tubes. Peripheral blood mononuclear cells (PBMCs) were separated from blood using Ficoll density gradient centrifugation (Lympholyte H, Cedarlane, CANADA).

Total RNA was extracted from PBMCs using Tripure isolation reagent (Roche, Germany) according to the manufacturer’s instructions. The quality and quantity of the extracted RNA were measured using a NanoDrop spectrophotometer. The mRNA was reverse transcribed using a RevertAid First Strand cDNA synthesis kit (Thermo Fisher Scientific, USA) according to the manufacturer’s instructions. 

***HTLV-1 proviral load***

To quantify the HTLV-I proviral load, PBMCs were isolated by Ficoll density gradient centrifugation (Lympholyte H, Cedarlane, CANADA), and a real-time PCR was performed using a commercial quantification kit (Novin Gene, Iran) by a Rotor-Gene Q-6000 real-time PCR machine (Qiagen, Germany) to measure the proviral load of HTLV-I in PBMCs. Albumin was used as an internal control. The normalized value of HTLV-1 proviral load was calculated via the following formula: (HTLV-1 DNA copies number/albumin DNA copies number/2) × 10^4^ and expressed as copies of HTLV-1 proviruses per 10^4 ^PBMCs (19-21).

***Quantitative real-time PCR***

Gene expression of IL-32 and COX-2 was evaluated with TaqMan real-time PCR assay. Glyceraldehyde-3-phosphate dehydrogenase (GAPDH) was used as a housekeeping gene for data normalization. Primers and probes used for detecting the desired genes are represented in Table 1.

***Enzyme-linked immunosorbent assay (ELISA) for IL-32***

Serum levels of IL-32 were evaluated by ELISA according to the manufacturer’s instructions (Bioassay Technology Laboratory, China).

***Statistical analysis***

Results were statistically analyzed in GraphPad Prism (version 7.0, GraphPad Software Inc., San Diego, CA, USA) and presented as means ± standard error of the mean (SEM). Differences in mean values between study groups were analyzed with One-way ANOVA and Kruskal–Wallis tests. The relationship between different variables was assessed using Spearman’s correlation analysis. *P*-values<0.05 were considered to be statistically significant. 

## Results

***Study population***

This study investigated 21 HAM/TSP patients (mean age 47.38 ± 12.67 years), 21 ACs (mean age 46 ± 10.82 years), and 21 HCs (mean age 47.38 ± 12.49 years). In each group, 19 out of 21 individuals were female (90%). No significant differences were observed between age and gender in study groups (*P*=0.999).

***Elevated IL-32 gene expression among HTLV-1 asymptomatic carriers***

**Figure 1:**
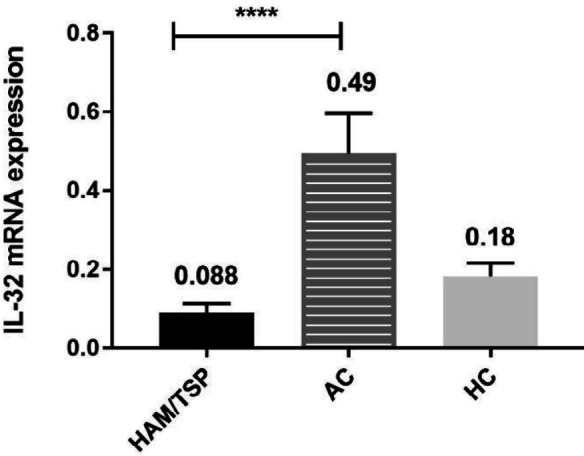
mRNA expression of IL-32 in HAM/TSP patients, ACs, and HCs. Results are presented as mean±SEM. *P<0.05, ** *P*<0.01, *** *P*<0.001, and **** *P*<0.0001; significant difference between groups

The mean IL-32 gene expression in ACs (0.49 ± 0.1) was significantly higher than in HAM/TSP patients (0.088 ± 0.024, *P*<0.0001). It was also shown that IL-32 mRNA levels were higher in ACs compared with HCs (0.18 ± 0.032). However, this was not a significant difference (*P*>0.05) (Figure 1).

***ELISA for IL-32***

**Figure 2:**
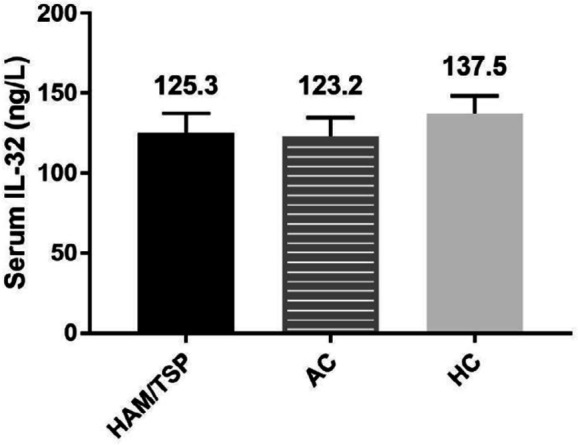
Serum levels of IL-32 in HAM/TSP patients, ACs, and HCs. Results are presented as mean ± SEM

ELISA assay was used to measure the serum levels of IL-32 in each group. Results indicate that the mean serum levels of IL-32 in HAM/TSP patients, ACs, and HCs were 125.3 ± 12.1 ng/l, 123.2 ± 11.5 ng/l, and 137.5 ± 10.8 ng/l, respectively. However, no statistically significant differences were observed among the three groups (*P*>0.05) (Figure 2).

***COX-2 mRNA expression***

**Figure 3:**
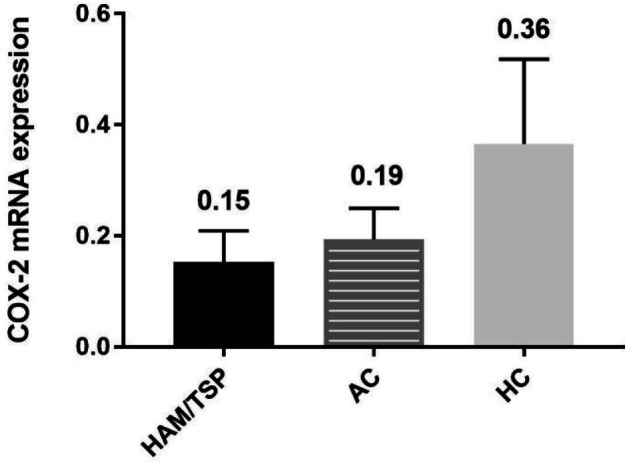
mRNA expression of COX-2 in HAM/TSP patients, ACs, and HCs. Results are presented as mean±SEM

The mean expression levels of COX-2 in HAM/TSP patients, ACs, and HCs were 0.15 ± 0.05, 0.19 ± 0.05, and 0.36 ± 0.15, respectively. There were no significant differences among the three groups (*P*>0.05) (Figure 3).

***HTLV–I proviral load***

The average HTLV-I proviral load was significantly higher in the HAM/TSP group compared with the ACs (3871 ± 512 copies/10^4^ PBMCs vs 1441 ± 285 copies/10^4^ PBMCs, respectively; *P*=0.0004).

Pearson’s correlation analysis revealed a significant negative correlation between the expression of IL-32 and HTLV-1 proviral load (*P*=0.07, R = -0.3). 

**Table 1:** The nucleotide sequence of primers and probes for IL-32 and COX-2

**Name**	**Primer name**	**Sequence (5'→3')**	**Product length(bp)**
	Forward	GACAGTGGCGGCTTATTATGAG	
IL-32	Reverse	TCCTCAACA TCCGGGACAG	114
	Probe	CTGTTGCCTCGGCACCGTAATCCATCT	
	Forward	CCGCAGTACAGA AAGTATCACAG	
COX-2	Reverse	GCAGACATTTCCT TTTCTCCTGTA	142
	Probe	CTTCCATTGACCAG AGCAGGCAGATGA	

HAM/TSP: HTLV-1 associated myelopathy/tropical spastic paraparesis ACs: Asymptomatic carriers; HCs: Healthy controls

HAM/TSP: HTLV-1 associated myelopathy/tropical spastic paraparesis ACs: Asymptomatic carriers; HCs: Healthy controls

HAM/TSP: HTLV-1 associated myelopathy/tropical spastic paraparesis ACs: Asymptomatic carriers; HCs: Healthy controls

## Discussion

In the present study, the IL-32 mRNA and protein levels as a newly discovered proinflammatory cytokine, COX-2 gene expression as an important factor in inflammation, and also HTLV-1 proviral load were evaluated in HAM/TSP patients and ACs. Furthermore, the correlation among these three factors was also assessed. 

Results obtained in the present study revealed elevated mRNA levels of IL-32 in ACs in comparison with HCs and HAM/TSP patients. However, this difference was statistically significant only between the ACs group and HAM/TSP patients. There were no statistically significant differences in the serum levels of IL-32 between the study groups. HAM/TSP patients showed a higher level of proviral load in comparison with ACs. Furthermore, we found a negative correlation between proviral load and IL-32 mRNA expression, by which in HAM/TSP patients elevated proviral load was accompanied by reduced IL-32 mRNA levels, while lower proviral load in ACs was associated with increased IL-32 gene expression. 

Previous studies have shown that IL-32 induces production of several inflammatory cytokines. It was reported that expression of IL-32 is associated with expression of proinflammatory cytokines such as IL-1β and IL-18 (22). IL-32 participates in inflammatory diseases such as rheumatoid arthritis (23, 24). Infectious agents such as *Mycobacterium tuberculosis*, EBV, HIV, HBV, HCV, and H1N1 were implicated in inducing the production of IL-32 (11, 12, 14, 23, 25-27). Contrary to the findings of our study it was reported that in chronic HCV infection, high levels of IL-32 support hepatic inflammation and liver fibrosis. Moreover, HCV and proinflammatory cytokines such as IL-1β and TNF-α up-regulate IL-32 production (14). 

In HIV-1 infection, IL-32 inhibits antiviral immune response and therefore supports HIV-1 replication, resulting in viral persistence. However, in the present study, high IL-32 mRNA levels in ACs were accompanied by low HTLV-1 PVL, and IL-32 mRNA levels were lower in HAM/TSP patients compared with ACs while the HAM/TSP patients had higher PVL (12, 28). On the other hand, depletion of endogenous IL-32 in HIV-1 infection resulted in down-regulation of proinflammatory cytokines and increased HIV-1 production (29). Therefore, IL-32 can also be an HIV-1 inhibitor. Hence, it could be concluded that IL-32 may be an HTLV-1 inhibitor that seems to inhibit virus replication in ACs, while in HAM/TSP patients with low mRNA levels of IL-32, inhibitory effects of IL-32 on HTLV-1 replication is diminished. Gene expression regulation can occur at the level of transcription or post-transcription, causing an imbalance between mRNA and protein levels. Therefore, a large amount of mRNA transcript might be observed in nuclear foci, before mRNA undergoes degradation in these foci (30). Thus, the elevated levels of IL-32 mRNA compared with low or normal protein levels in ACs might be associated with regulation of gene expression at different levels.

There are more than nine isoforms of IL-32 which are transformed into four major splice variants including IL-32α, IL-32β, IL-32γ, and IL-32δ, due to multiple mRNA splicing (31). In the present study, we evaluated the total IL-32 with the ELISA assay. Considering the antiviral activity of IL-32γ (32), it is recommended that future studies investigate the levels of IL-32γ in HAM/TSP patients. 

COX-2 is a proinflammatory factor and a critical mediator of inflammatory pathways (33, 34). Most viral infections induce COX-2 production (35). Influenza A virus induces the production of COX-2 and IL-32. In fact, in infection with influenza A, COX-2 triggers IL-32 production, and high IL-32 levels conversely inhibit COX-2 function (36). However, in EBV infection it was shown that COX-2 accumulation in PBMCs was reduced (37). In the present study, the levels of COX-2 in HAM/TSP patients were more than in ACs and HCs. However, this was not a significant difference.

## Conclusion

Based on our findings, IL-32 seems to be negatively correlated with HTLV-1 proviral load, by which high levels of IL-32 inhibit HTLV-1 replication, and therefore, IL-32 could be a natural HTLV-1 inhibitor. However, a clear relationship between COX-2, HTLV-1, and IL-32 was not observed in this study. Several factors could influence the outcome of HTLV-1 infection and it is difficult to find a clear relationship between these factors and HTLV-1 pathogenesis. Therefore, we need to continue the effort by conducting further studies with a larger sample size to understand the role of COX-2 and IL-32 in HTLV-1 pathogenesis.
